# Dental root canal treatment complicated by foreign body ingestion: a case report

**DOI:** 10.1186/1757-1626-2-117

**Published:** 2009-02-03

**Authors:** Ramyia G Dhandapani, Susim Kumar, Mark E O'Donnell, Ted McNaboe, Brian Cranley, Geoff Blake

**Affiliations:** 1 Department of General Surgery, Daisy Hill Hospital, Newry BT35 8DR, Northern Ireland, UK; 2 Department of Ear, Nose and Throat, Daisy Hill Hospital, Newry BT35 8DR, Northern Ireland, UK; 3 School of Health Sciences, University of Ulster, Jordanstown Campus, Shore Rd, Newtownabbey BT37 0QB, Northern Ireland, UK

## Abstract

**Introduction:**

Most foreign bodies pass through the gastrointestinal tract uneventfully. The majority of the reported literature describes the management of ingested blunt objects. However, ingestion of sharp objects can still occur with a higher rate of perforation corresponding to treatment dilemmas.

**Case Presentation:**

We report the conservative management of an inadvertently ingested sharp foreign body during a routine dental procedure and describe a management strategy for the treatment of both blunt and sharp foreign bodies.

**Conclusion:**

Urgent endoscopic assessment and retrieval is indicated when there is a history of a recently ingested sharp foreign body or if clinical suspicion suggests that the object is located within the oesophagus. Conservative management is advocated if the object has passed through the pylorus with serial clinical assessments including daily radiographs. Surgical intervention is warranted in the presence of obstruction, perforation or peritonitis.

## Background

Accidental foreign body ingestion is a common clinical problem especially in children. Ingestion still occurs in adults but is often identified in elderly, mentally impaired or patients with alcohol dependency. Intentional foreign body ingestion may also be experienced in prisoners or psychiatric patients.[1] Although complications are higher with sharp implements, reported rates of gastrointestinal perforation still remain rare at less than 1%.[1-3] Dentures and small orthodontic appliances (73%) account for the majority of accidental sharp ingestion in normal adults.[4] Other commonly ingested sharp objects also include sewing needles, tooth picks, chicken and fish bones, straightened paper clips and razor blades.[5,6]

## Case presentation

**Figure 1:**
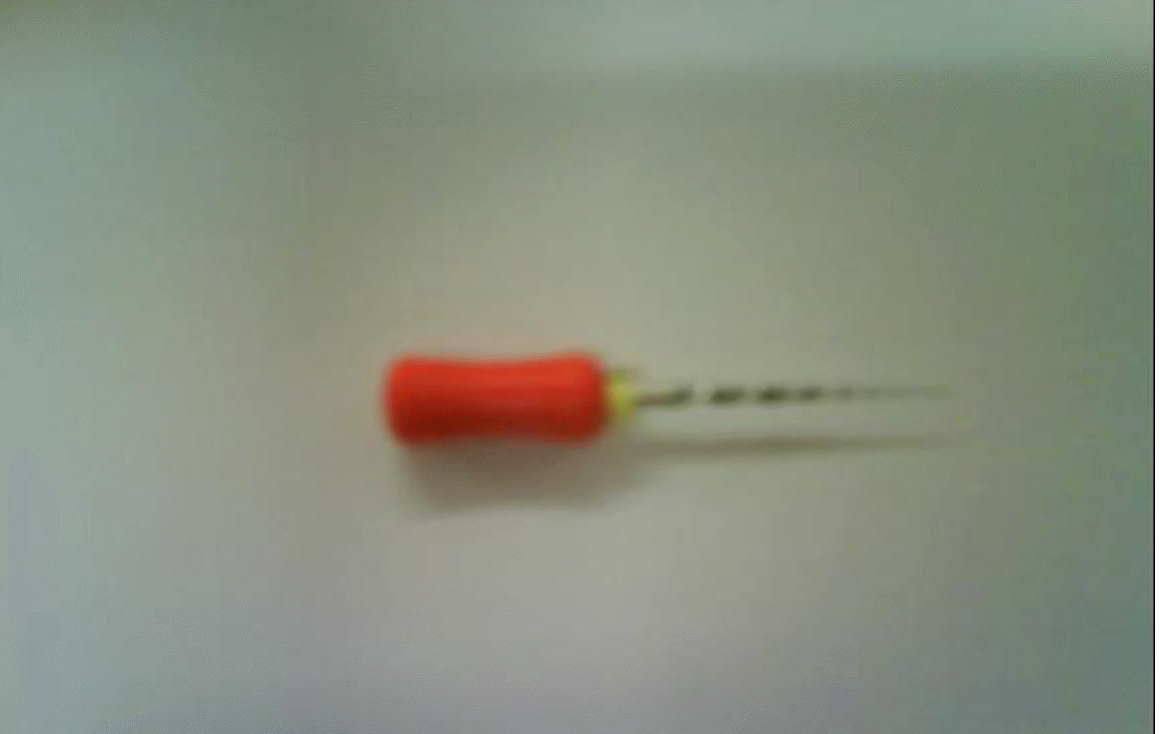
**Root canal (Endodontic) file (Dent Supply, BF Mulholland Ltd, Glenavy, Northern Ireland)**.

A 36-year old man presented to the Emergency Department following the accidental ingestion of an endodontic file during a routine root canal dental procedure (Figure 1). The patient complained of excessive gagging along with the sensation of "something sticking in his throat". There was no history of nausea, vomiting or abdominal pain. On examination he was haemodynamically stable with no evidence of airway compromise, respiratory distress or abdominal tenderness. An ENT assessment was normal suggesting passage of the foreign body into the oesophagus.

**Figure 2:**
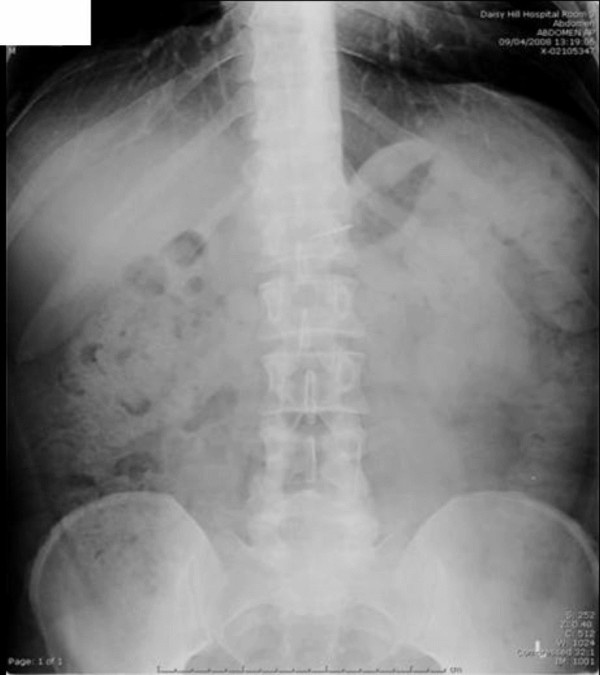
**Abdominal X-ray (AXR) demonstrating root canal file at the level of the L1 vertebra**.

A plain abdominal x-ray demonstrated the presence of a sharp foreign body overlying the pyloric region at the level of the L1 vertebral body (Figure 2). An erect chest x-ray was normal. Urgent oesophago-gastro-duodenoscopy failed to retrieve the foreign body.

**Figure 3:**
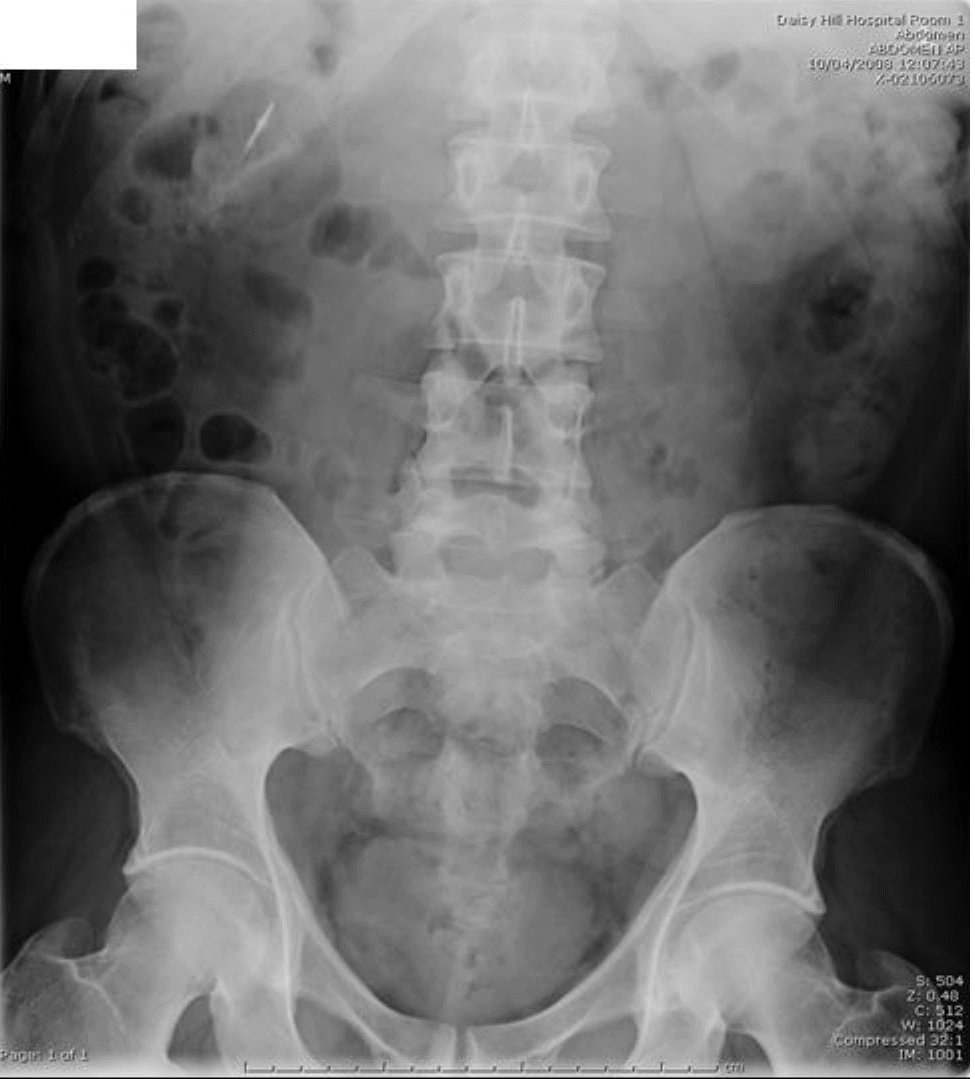
**AXR on day 2 – Sharp foreign body has progressed to the hepatic flexure**.

**Figure 4:**
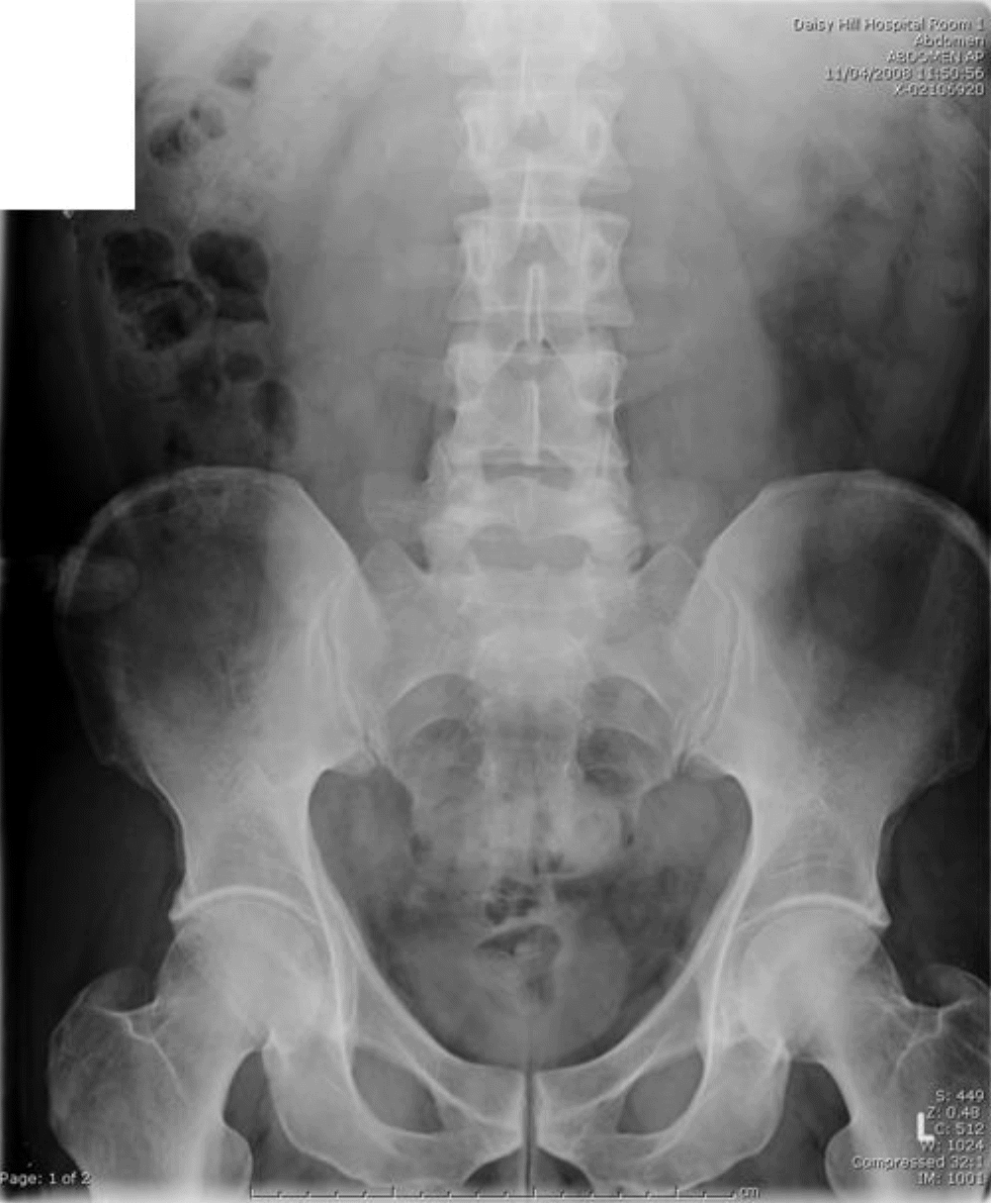
**AXR on day 3 – showing no further evidence of the FB**.

The patient was treated conservatively and kept nil by mouth for 24-hours with regular observations. He remained well the following day with no clinical evidence of intestinal obstruction or perforation. Normal diet was commenced. Serial abdominal X-rays showed passage of the foreign body through the hepatic flexure on day-2 (Figure 3) and complete passage through the gastrointestinal tract by day three (Figure 4). He was subsequently discharged with no further follow-up.

## Discussion

Ninety percent of ingested foreign bodies pass through the gastrointestinal tract uneventfully. Endodontic files have been previously reported to pass out through the gastro-intestinal system within 3-days without incident.[7] Approximately 10% necessitate endoscopic removal while only 1% will ever require surgical intervention. Impaction may occur at sites of anatomical or physiological narrowing such as the lower oesophageal sphincter, ileocaecal valve or in areas of stricture formation. If the object has passed into the stomach and is less than 6 cm in length and 2 cm in diameter, there is a 90% chance of spontaneous passage through the pylorus and ileocaecal valve.[1,5,6,8,9] Patients with previous gastro-intestinal tract surgery or congenital gut malformations are at increased risk of perforation.[5] With sharp objects, the most common sites of perforation are the lower oesophagus and terminal ileum.[5,9] Perforation is caused by direct penetration or pressure necrosis due to prolonged lodgement. The subsequent foreign body migration can lead to abscess or fistulae formation, which can present a diagnostic challenge in late presentations.[10]

Radio-opaque foreign bodies are most commonly identified with plain radiographs. Other investigative modalities include ultrasound scanning, computerised tomography or magnetic resonance imaging. Contrast studies are not routinely indicated owing to the risk of aspiration. Upper and lower gastrointestinal tract endoscopy can be used diagnostically or therapeutically. Passage of a sharp foreign body into the stomach or duodenum still requires immediate attempts at endoscopic retrieval, as the risk of perforation on reaching the ileocaecal valve is approximately 35%.[1,5,9] Endoscopic retrieval in these situations has a success rate of 86% and complications occur in less than 2% of cases.[5] Attempts at endoscopic extraction of foreign bodies such as partial dentures, can lead to laceration of the oesophagus, escalating to mediastinitis, pneumothorax, and pneumopericardium; a flexible endoscope fitted with a latex hood can facilitate matters[11]. Detection of an impacted foreign body, for example a dental prosthesis, in the colon, is commonly delayed until complications such as perforation or abscess formation evolve.[12] Such objects can be successfully removed colonoscopically.[12]

If a sharp object has progressed beyond the duodenum or endoscopy has proved unsuccessful in retrieving the object, the patient should remain under strict observation with daily radiographs. Progressively deteriorating symptomatology or systemic sepsis may often require either laparoscopic or open surgical intervention [4]. Table 1 documents a recommended management protocol for the treatment of ingested sharp and blunt foreign bodies.

**Table 1:** Recommended management protocol for the treatment of ingested sharp and blunt foreign bodies (*adapted from Bisharat et al. 2007*).

Type of Object	Site of Object	Management Protocol
Sharp Metallic Objects	Oesophagus	Urgent endoscopic retrieval
	Stomach and Duodenum	Urgent endoscopic retrieval
	> DJ Flexure	Daily x-rays/strict observation
		If fails to progress > 72 hrs → laparotomy
		If signs of Obstruction/Bleeding/Perforation → laparotomy
		
Blunt Metallic Objects	Oesophagus	Endoscopic retrieval
	Stomach and Duodenum	If < 2 cm → weekly X-rays/conservative management
		If > 2 cm → observe with weekly X-rays for 1–2 months. If failure to progress → endoscopic retrieval
	> DJ Flexure	Weekly X-rays/conservative management
		If signs of Obstruction/Bleeding/Perforation → urgent endoscopic retrieval +/- laparotomy

## Conclusion

Urgent endoscopic assessment and retrieval is indicated when there is a history of a recently ingested sharp foreign body or if clinical suspicion suggests that the object is located within the oesophagus. Conservative management is advocated if the object has passed through the pylorus with serial clinical assessments including daily radiographs. Surgical intervention is warranted in the presence of obstruction, perforation or peritonitis.

## Competing interests

The authors declare that they have no competing interests.

## Authors' contributions

All authors have read and approved the final manuscript. RGD: Involved in the literature review and manuscript preparation. SK: Involved in the literature review, manuscript preparation and manuscript editing. MEOD: Involved in the conception of the report, literature review, manuscript preparation, manuscript editing, and manuscript submission. TMN: Involved in manuscript editing and manuscript review. BC: Involved in manuscript editing and manuscript review. GB: Involved in the manuscript editing and manuscript review.

## Consent

Written informed patient consent was obtained from both patients for the publication of this study. No source of funding has been declared by the authors.
